# Anthropogenic disturbances are key to maintaining the biodiversity of grasslands

**DOI:** 10.1038/srep22132

**Published:** 2016-02-23

**Authors:** Z. Y. Yuan, F. Jiao, Y. H. Li, Robert L. Kallenbach

**Affiliations:** 1 State Key Laboratory of Soil Erosion and Dryland Farming on the Loess Plateau, Institute of Soil and Water Conservation, Northwest A&F University, Yangling, Shaanxi 712100, PR China; 2 Institute of Soil and Water Conservation, Chinese Academy of Science and Ministry of Water Resource, Yangling, Shaanxi 712100, China; 3 Institute of Grassland Research, Chinese Academy of Agricultural Sciences, Hohhot, Inner Mongolia, 010010, PR China; 4 University of Missouri, Division of Plant Sciences, 108 Waters Hall, Columbia, MO 65211, USA

## Abstract

Although anthropogenic disturbances are often perceived as detrimental to plant biodiversity, the relationship between biodiversity and disturbance remains unclear. Opinions diverge on how natural diversity is generated and maintained. We conducted a large-scale investigation of a temperate grassland system in Inner Mongolia and assessed the richness-disturbance relationship using grazing intensity, the primary anthropogenic disturbance in the region. Vascular plant-species richness peaked at an intermediate level of anthropogenic disturbance. Our results support the Intermediate Disturbance Hypothesis, which provides a valid and useful measure of biodiversity at a metacommunity scale, indicating that anthropogenic disturbances are necessary to conserve the biodiversity of grassland systems.

Anthropogenic disturbances often cause habitat loss, ecological fragmentation, and loss of biodiversity^1^. Ecological models and public policies put a heavy emphasis on the negative effects of grazing but often fail to acknowledge the potentially positive effects of grazing on biodiversity in grassland ecosystems. The Intermediate Disturbance Hypothesis (IDH) proposes that within a broad range of environmental disturbance levels, species diversity is maximized at an intermediate level of anthropogenic and natural disturbances, because competitively inferior, disturbance-tolerant species and competitively dominant, disturbance-sensitive species coexist when disturbances are neither too rare nor too frequent^2^,^3^. With low levels of disturbance, richness is predicted to be low because of competitive exclusion. With high levels of disturbance, richness is predicted to be low, because most species cannot tolerate frequent destructive events. With intermediate levels of disturbance, richness is predicted to be high, however, because dominant competitors and rapid colonizers are able to coexist^4^,^5^.

Connell^3^ first introduced the IDH with supporting data from tropical rain forests and coral reefs in 1978. Many ecology textbooks feature the IDH. Connell’s original paper on the topic has received more than 4,000 citations in the past 40 years and is still referenced in important scientific papers at an increasing rate, according to ISI Web of Science. The IDH has been extensively validated with both observational and experimental data^6^,^7^,^8^,^9^,^10^. Although the IDH is widely used to explain species diversity patterns, the considerable circumstantial evidence gathered in the past 40 years both for and against the IDH has led to controversy^11^,^12^,^13^,^14^,^15^. Debate about the IDH has led to healthy discussions in the scientific community and prompted investigators to assess experimental designs before collecting data, as Kallenbach^16^ proposed, in order to better evaluate scientific models.

An increasing number of critiques reject the IDH as an explanation of spatial patterns of species diversity. Recently, Fox^11^ argued that the IDH fails on both empirical and theoretical grounds and so should be absolutely abandoned. In contrast, Huston^15^ stated that Fox made fundamental errors, creating oversimplified caricatures of the IDH by ignoring all of the coexistence-promoting mechanisms discussed by Connell^3^. The controversy exemplifies the limits to our ability to predict ecosystem responses to human disturbance. We still do not know whether the IDH represents an important mechanism of species coexistence in the real world. Furthermore, the recent arguments about the IDH^11^,^15^ highlight the importance of refining how we develop and test ecological theories.

The IDH model suggests that any community can reach maximum diversity through multiple mechanisms^5^,^12^, which can be described biologically or mathematically and might vary with locality and trophic level^4^,^17^. One general assumption of the IDH is that trade-offs between competitive ability and colonization ability facilitate the disturbance-mediated coexistence of competitively superior species, which are unable to thrive in highly disturbed sites, and colonizer species, which can be outcompeted by competitively superior species in less-disturbed sites^12^,^18^.

Competitively superior and colonizer species can, however, coexist between those extremes, leading to higher biodiversity at intermediate disturbance levels^3^,^12^. Factors such as species’ ability to utilize and partition resources, often reflected in productivity, are involved in the mechanisms that promote species coexistence^12^,^19^. As Kallenbach^20^ explained, plants such as tall fescue (*Lolium arundinaceae* L.) that form endophytic relationships with fungi are more able to persist in the face of harsh environmental conditions and overgrazing than plants that do not form endophytic relationships. Tall fescue thus gains a competitive advantage in certain ecosystems, which disturbance models must account for. Alternative theoretical models, including the ‘storage effect’^5^ and ‘successional niche’^21^ models, suggest that coexistence can occur when disturbances create spatiotemporal niches in which competitively inferior species gain novel competitive advantages over otherwise competitively dominant species.

Species richness varies with spatial scale^22^,^23^,^24^,^25^,^26^, suggesting that competition and other density-dependent processes might be unimportant to determining patterns of species richness at certain spatial scales. Many reports that are critical of the IDH tested the diversity-disturbance relationships across multiple biomes^27^ or, more commonly, at small spatial scales within communities^28^,^29^,^30^. Compared with anthropogenic disturbances, other biotic and abiotic factors that impact diversity at the regional scale change slowly. Sampling vegetation at the regional scale therefore provides an ideal opportunity to test the IDH. The IDH was originally developed for coral reefs and tropical rain forests and was later found to apply to other terrestrial and aquatic (marine) systems with only natural disturbances^31^,^32^,^33^. Some studies have tested the diversity patterns in arid and semi-arid grasslands^27^,^34^,^35^, but often on a large scale over multiple biomes or on a small scale within communities. Few studies have tested the diversity pattern predicted by the IDH at the regional scale.

We surveyed the vascular plant species near 100 local community plots scattered across 62,500 km^2^ of arid grasslands in the Inner Mongolian area of northern China (Supplementary materials, Google Earth KMZ file)^36^ and tested whether species diversity displays a normally distributed bell curve to disturbances, as the IDH predicts. The local communities of trophically interacting species occupy discrete resource patches linked by dispersal and can therefore be viewed as a metacommunity^37^,^38^ i.e., a set of interacting local communities which are linked by the dispersal of multiple, potentially interacting species. Because the primary anthropogenic disturbance in the region is livestock (i.e., sheep and goat) grazing, we used the grazing intensity within a 5 km radius of each community as a direct measure of anthropogenic disturbance. The grazing intensity was greatest near the most populous towns and least in a natural reserve, where livestock had been excluded for 35 years. The relationship between species richness and disturbance level was unimodal with a peak at an intermediate level of disturbance, supporting the IDH.

## Results

**Figure 1:**
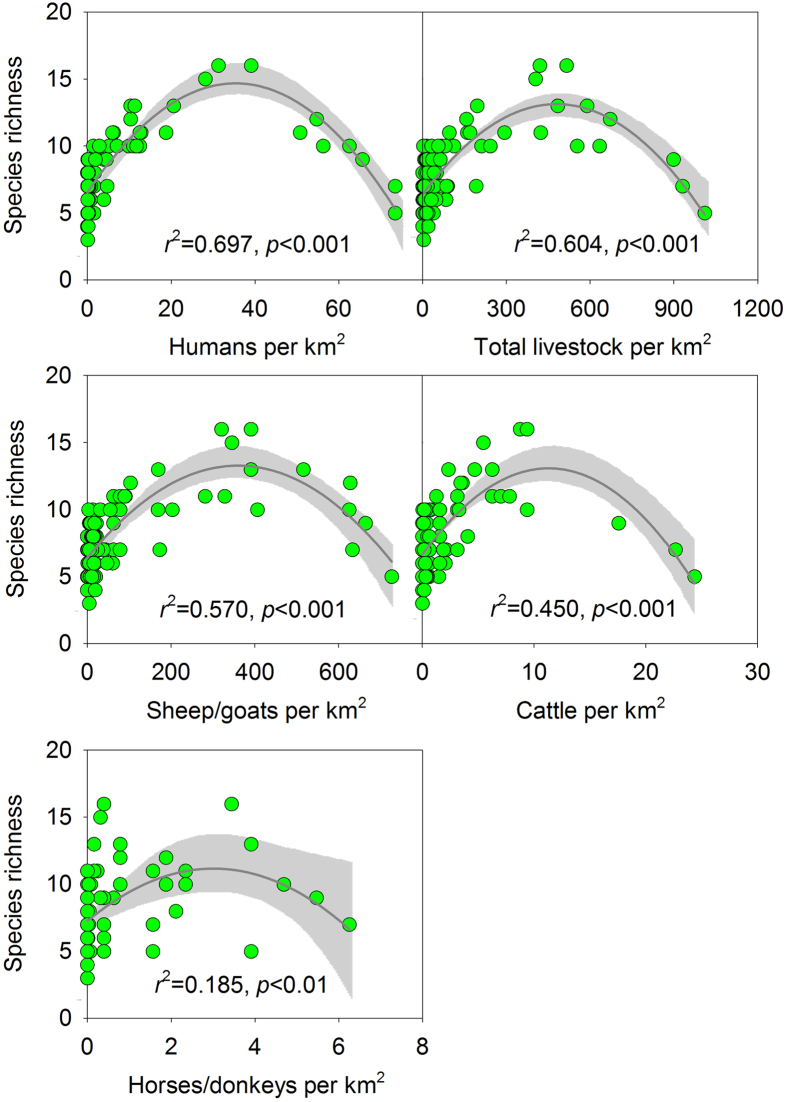
Relationships between species richness and disturbance. The relationships are best described by non-linear regression (quadratic models, dark grey lines). Grey shade refers to Loess smoothing with 95% confidence intervals.

Unimodal curves best described the relationships between the vascular plant-species richness and the human disturbance level (Fig. 1). Although we found similar diversity-disturbance trends when we expressed the species diversity using the Shannon-Weiner index and the Simpson index (Supplementary materials, Figs S1 and S2), both the Shannon-Weiner index and the Simpson index had lower R-square values than the richness index. Neither the mean annual temperature nor the precipitation was significantly associated with the species richness in a Poisson regression model. In contrast, both the human population size and the intensity of livestock disturbance were positively and significantly associated with the species richness (Table 1). The species richness peaked at a human population density of 35 individuals per km^2^. A simple quadratic curve (*r*^2^ = 0.570, *p* < 0.001) provided the best fit for the relationship between species richness and the major livestock disturbance in the region, grazing by sheep and goats. The unimodal model revealed that the species richness peaked at the stocking rate of 360 sheep/goats km^−2^. Simple unimodal models also provided the best description of the vascular plant-species richness in relation to other livestock-related disturbances, including those from cattle and horses. When the total grazing intensity from all livestock was considered, a simple quadratic curve (*r*^2^ = 0.604, *p* < 0.001) demonstrated that species richness peaked at 480 animals km^−2^ (see the calculation in the Methods). The livestock disturbance attributed to the sheep and goats was a more accurate predictor of species richness than that attributed to the cattle and horses.

In general, the plant species diversity was significantly associated with the human population size and the livestock disturbance but not with the climate (Table 2). The climate data (mean annual temperature and precipitation, MAT and MAP, respectively) collectively explained 17% of the variation in species richness. The climate data combined with the human population size and the livestock disturbance explained 62% of the variation in species richness. Thus, the climate and the level of anthropogenic disturbance, particularly the latter, made a major contribution to the observed variation in species richness. The climate and anthropogenic disturbance level contributed similar fractions to the Shannon-Weiner index and the Simpson index (Table 2).

**Figure 2:**
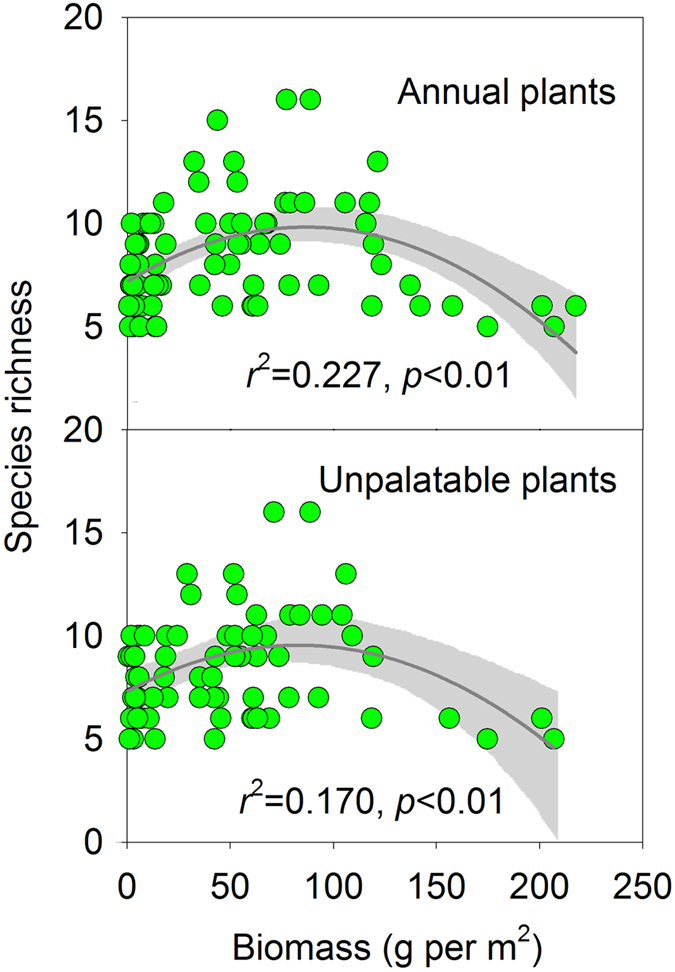
Relationships between species richness and biomass of annual and unpalatable plants. The relationships are best described by non-linear regression (quadratic models, dark grey lines). Grey shade refers to Loess smoothing with 95% confidence intervals.

In accordance with their life cycles, annual plant species often occupy recently disturbed areas^39^. Therefore, the biomass of the annual vascular plants should reflect the disturbance intensity. We found that species richness peaked at an intermediate level of annual plant biomass (*r*^2^ = 0.227, *p* < 0.001). There was a simple unimodal relationship between the species richness and the biomass of unpalatable species like *Artemisia annua* L. and *A. sieversiana* L. (Fig. 2). Both of those species are unpalatable to livestock and tend to dominate the recently disturbed patches of the study region.

## Discussion

Overgrazing by livestock on the grasslands of Inner Mongolia has become an important issue over the past several decades^40^,^41^. We found a unimodal relationship between the vascular plant-species richness and disturbance by herbivores in Inner Mongolian grassland systems, in contrast to other studies that did not observe a diversity peak at intermediate disturbance levels^11^,^42^. Our results support the IDH and reinforce the view that anthropogenic disturbance cannot be abandoned as a regulatory force in species structuring^15^.

Our survey covered multiple levels of a wide disturbance gradient (i.e., grazing intensity)^36^,^43^. In contrast, the narrow range of disturbances examined in many previous studies could have missed the species diversity peak at intermediate disturbance levels by collecting data at disturbance levels either too small or too great to capture the full range of responses. When Connell^3^ first proposed the IDH, his evidence came from a Ugandan forest that followed a successional sequence gradient from no disturbance (low diversity) to intensified disturbance (high diversity). Testing the IDH requires a detection system with sufficient sampling to cover both the rising and the falling sections of the curve.

Connell^3^ suggested that the IDH “deals only with variations in diversity within local areas”. The “local areas” do not refer to “large-scale geographical gradients such as tropical to temperate differences” or to sites within small communities, but rather to intermediate sized areas on a regional scale. Presumably the 35,200 ha Budongo rainforest^44^ that Connell used as reference data could be called a metacommunity, which is composed of local communities^37^,^45^. Unfortunately, studies to test the IDH often make their conclusions based on large-scale sampling, such as sampling on a global scale^27^, or on inadequate, small-scale sampling^11^,^42^,^46^ (<100 ha). Hence, results from the IDH model are often contradictory and might be related to the scales used in the various studies.

Although we did not make a scale-related comparison in this study, our results at the metacommunity scale suggest that the results of other studies conducted at global or local scales might be attributed to the size of the sampling area. Diversity-related processes at smaller scales, such as those within a single community, or at larger scales, such as those across biomes, might differ as disturbance mechanisms provide different outcomes as a function of scale^15^,^47^,^48^,^49^. Intra-community observations cannot capture variation in species richness, whereas inter-biome observations cannot detect the richness-disturbance pattern predicted by the IDH. We collected all of our samples under similar climatic and soil conditions. Furthermore, we minimized the impacts of other coexistence mechanisms that factor into larger spatial scales.

The logic of the IDH is not wrong. The supposed empirical failure of the IDH might be due to a failure to consider the conditions under which the IDH is valid. The unimodal (quadratic), intermediate disturbance pattern can only be detected within a specific range of productivity and mortality conditions^15^,^50^. If data are collected along a disturbance gradient from sites that differ greatly in productivity, the effect of the disturbance on diversity might be completely obscured. As the Dynamic Equilibrium Model predicts^51^, the level of productivity would control the effects of disturbance on diversity.

The goal of the IDH is not to demonstrate the long-term stable coexistence of species under the equilibrium conditions of mathematical theory, but rather to explain or predict patterns of species diversity that can be measured. There are so many mechanisms involved in coexistence that it is impossible to reveal whether long-term stable coexistence of species occurs in the real world^15^. The richness-disturbance pattern observed in our study, as predicted by the IDH, suggests that we still need to figure out the underlying mechanisms that contribute to competition, coexistence, and speciation over different spatial and temporal scales.

For appropriate tests of the IDH, it is important to use appropriate measures of disturbance and diversity^9^. Connell did not address how to define or measure disturbance regimes. In the Inner Mongolian grassland ecosystems, domestic livestock grazing is the primary anthropogenic disturbance that shapes species diversity and productivity. The grazing intensity is a direct measure of disturbance and is better than the indirect indices used in most studies, such as those for vegetation cover, canopy height, species density of pioneer trees, heliophilic stems, and basal area^31^,^32^,^52^,^53^,^54^. Frequency, intensity, extent, duration, and time since last disturbance^12^ are common terms used to describe an environmental disturbance. Based on our observations, grazing intensity was the dominant anthropogenic disturbance. The sites in our study differed in disturbance intensity but were similar in many other attributes such as climate and soil.

We found that human encroachment favored some species over others; the recently disturbed patches were dominated by annual *Artemisia* species that livestock find unpalatable. The species diversity within the region was unimodally related to the biomass of the annual *Artemisia* species, suggesting that those species play a role in species coexistence mechanisms (Figs S3 and S4). The undisturbed sites might have lower diversity because the dominant species inhibit the establishment and succession of less-fit species through competitive exclusion^55^.

The observed, unimodal diversity-disturbance relationship in our study suggests that certain prerequisites of the IDH; such as competitive exclusion, successional stages, and trade-offs between competition and tolerance^3^,^56^; occur along the grazing gradient in the studied sites. Our findings also indicate that the vegetation in the arid regions of Inner Mongolia is not resistant to grazing, and that the effects of grazing on species diversity vary as a function of the condition of the landscape (i.e., environmental stress).

The human population density explained more of the diversity than the livestock population density. Why that should be the case is unclear, but one possibility is that the human population causes disturbances not only through livestock grazing but also through trampling, fires, digging for medical herbs, the collection of grassland products, and the establishment of camps and roads. All of those activities can cause physical, and hence ecological, disturbances to grassland areas, potentially influencing diversity.

Although the various methods to estimate diversity introduced in the literature might respond differently to disturbances^9^,^42^, we found similar diversity-disturbance relationships for the species richness, Shannon-Weiner, and Simpson indices. The species richness index had the highest R-square values, suggesting that measure is more relevant to the predictions of the IDH than the Shannon-Weiner index, which was in turn better than the Simpson index. Our results suggested that the number of species estimated by the species richness index, rather than dominance estimated by the Shannon-Weiner and Simpon indices, is an appropriate response variable for the system in tests of the IDH. We could not determine the underlying mechanisms to explain the relationship between magnitude of disturbance and specific measures of biodiversity. However, our findings clearly indicate species richness is the important aspect of diversity and it changes in response to disturbance.

Our simple, unimodal, diversity-disturbance models for the typical grassland systems in Inner Mongolia explain how vascular plant-species richness varies with anthropogenic influences on a regional scale. Governmental agencies could use the models generated here to manage the biodiversity of the natural grasslands that cover more than 40% of the region^57^. Consistent with experimental studies of sheep grazing intensity^58^, our results revealed that vascular plant-species richness peaks at a moderate level of grazing intensity (480 sheep km^−2^). Thus the species richness of the grassland ecosystems of Inner Mongolia would be best conserved by properly managed grazing with an acceptable stocking rate rather than by the complete exclusion of livestock. The balanced coexistence of the plants and animals common to the region defines the ideal grassland system. Our results indicate that domesticated livestock grazing has excessively disturbed around 8% of the Inner Mongolian grasslands. Like grassland systems worldwide, those in Inner Mongolia can tolerate grazing disturbance, but only in moderation.

Although several scientists challenge the IDH^11^,^42^, our results suggest that the IDH provides a useful explanation of the response of biodiversity to disturbance at the regional (or metacommunity) scale. The IDH is one potential explanation of an observed unimodal pattern between species richness and disturbance. That does not exclude the involvement of other mechanisms, because climate and anthropogenic disturbances together explained only two-thirds of the variation in diversity in our study. The IDH suggests that disturbance contributes to biodiversity, although it might be difficult to detect the unimodal pattern in improperly scaled communities^31^,^59^. Our results indicate that the IDH is useful, albeit requiring greater precision in its definition. Many of the criticisms of the IDH are misguided, because they do not recognize the underlying logical assumptions of the hypothesis and consequently fail to test the hypothesis appropriately. The IDH holds in nature under specific conditions and thus remains a conceptually useful model. In an increasingly “disturbed” world^60^,^61^, many will find it ironic that the preservation of biodiversity depends on both encouraging and limiting disturbances within ecological thresholds.

## Methods

### Study area

The study area was situated in a typical temperate grassland near Xilin Guole (44°56′N, 115°22′E), Inner Mongolia, China. The region’s climate is arid and cold with an average temperature of 1.4 °C and precipitation of 301 mm annually (Supplementary materials, excel file). Most of the soils in the region are classified as dark chestnut (Mollisols) according to FAO system of soil classification. The study area has a long history of grazing by domestic livestock under nomadic or seminomadic patterns of land use. The types of livestock are sheep, goats, horses, cattle, and occasionally donkeys.

We selected 100 sites and surveyed the richness of vascular plant species within each site during August 2013. The sites were selected in a grid with grid center spacing ~10 km and were owned by herdsmen. The widely distributed sites represent more than 62,500 km^2^ of arid grasslands (Supplementary materials, Google Earth KMZ file). We assessed human disturbance by measuring the intensity of livestock grazing, the primary disturbance in the region. Natural disturbances such as fire and insect damage, especially the former, are not common in the region and were not considered as anthropogenic disturbances. To avoid missing rare species, we chose a ~1 ha sampling area at each of the 100 sites and randomly imposed three 5 × 5 m sampling plots at each sampling area. We identified and recorded all vascular plant species. We set up a 1 × 1 m subplot within each sampling plot and harvested the aboveground biomass. We investigated the intensity of livestock grazing by visiting herdsman and the related government Bureau of Animal Husbandry within a ~5 km radius around the sampling plots. Because different types of livestock consume different amounts of forage, we estimated the total effect of grazing disturbance by a standardized livestock number: total grazing intensity = (number of sheep × 1) + (number of goats × 0.9) + (number of cattle × 6) + (number of horses × 7) + (number of donkeys × 3)^62^,^63^.

### Data analysis

Species richness was expressed as the number of vascular plant species present within a plot. Species diversity was expressed by the Shannon-Weiner index [−(Σ*p*_*i*_ ln*p*_*i*_)] or by Simpson’s index [1/(Σ*p*_*i*_^2^)], where *p*_*i*_ is the proportion of total vascular plant cover contributed by species *i*. We performed linear and polynomial (quadratic and cubic) regression analyses to determine the best-fit shape of the species richness–disturbance relationship. The parameters of the Poisson regression were estimated using the R package *glmmADMB*. We also applied Loess smoothing using the R package *ggplot2* to assess possible nonlinearity between species richness and disturbance. All statistical analyses were performed using R 3.2.2.

## Additional Information

**How to cite this article**: Yuan, Z. Y. *et al.* Anthropogenic disturbances are key to maintaining the biodiversity of grasslands. *Sci. Rep.* **6**, 22132; doi: 10.1038/srep22132 (2016).

## Supplementary Material

Supplementary Information

## Figures and Tables

**Table 1:** Parameters estimated by standard Poisson regression.

Coefficient	Estimate	Std. error	z value	*p* > |z|
MAT	0.179	0.151	1.190	0.235
MAP	0.007	0.007	1.070	0.286
Human density	0.028	0.007	3.880	<0.001
Livestock intensity	0.001	<0.001	3.350	<0.001

MAT and MAP stand for mean annual temperature and precipitation, respectively.

**Table 2:** Whole-model *R* ^2^ values for multiple regression analyses of biomass and diversity in a series of models with increasing numbers of independent variables.

Model	Biomass	Richness	Shannon	Simpson
Latitude	0.024ns	0.067*	0.036ns	0.004ns
MAT	0.005ns	0.013ns	0.002ns	0.009ns
MAP	0.136***	0.131***	0.083*	0.047ns
MAT & MAP	0.168*	0.173*	0.143*	0.113ns
Human density	0.227***	0.562***	0.249***	0.142**
Livestock density	0.178***	0.497***	0.285***	0.179***
Human & livestock	0.267***	0.582***	0.263***	0.192*
Climate + disturbance	0.311***	0.623***	0.386***	0.355***

Overall model significance is: ns (not significant, *P* > 0.05), *(*P* < 0.05), **(*P* < 0.01) or ***(*P* < 0.001). The ‘Climate’ models include mean annual temperature (MAT) and mean annual precipitation (MAP). The ‘Disturbance’ models include densities of humans and livestock. The ‘Climate + disturbance’ models include the two sets combined.
