# Supplementary material for: Anthropogenic disturbances are key to maintaining the biodiversity of grasslands
Source: Sci Rep. 2016 Feb 23;6:22132. doi: 10.1038/srep22132 (PMC4763272; doi:10.1038/srep22132)

Supplementary Information for

Anthropogenic disturbances are key to maintaining the biodiversity of grasslands

Authors

Z.Y. Yuan[1, 2], F. Jiao[1,2*], Y.H. Li[3], Robert L. Kallenbach[4]

Corresponding to ZYY: zyyuan@ms.iswc.ac.cn

The PDF includes:

Additional captions for Figures 1 to 4

Supplementary Figures 1 to 4

Additional captions for Figures 1 to 4

Figure S1

Relationships between Shannon-Wiener index and disturbance. The relationships are best described by non-linear regression (dark grey lines). Nonlinear patterns are derived by Loess smoothing (grey shade) with 95% confidence intervals.

Figure S2

Relationships between Shannon-Wiener index and disturbance. The relationships are best described by non-linear regression (dark grey lines). Nonlinear patterns are derived by Loess smoothing (grey shade) with 95% confidence intervals.

Figure S3

Relationships between annual plant biomass and disturbance. The relationships are best described by non-linear regression (dark grey lines). Nonlinear patterns are derived by Loess smoothing (grey shade) with 95% confidence intervals.

Figure S4

Relationships between unpalatable plant biomass and disturbance. The relationships are best described by non-linear regression (dark grey lines). Nonlinear patterns are derived by Loess smoothing (grey shade) with 95% confidence intervals.

Supplementary Figures 1 to 4

Figure S1

Relationships between Shannon-Wiener index and disturbance. The relationships are best described by non-linear regression (dark grey lines). Nonlinear patterns are derived by Loess smoothing (grey shade) with 95% confidence intervals.

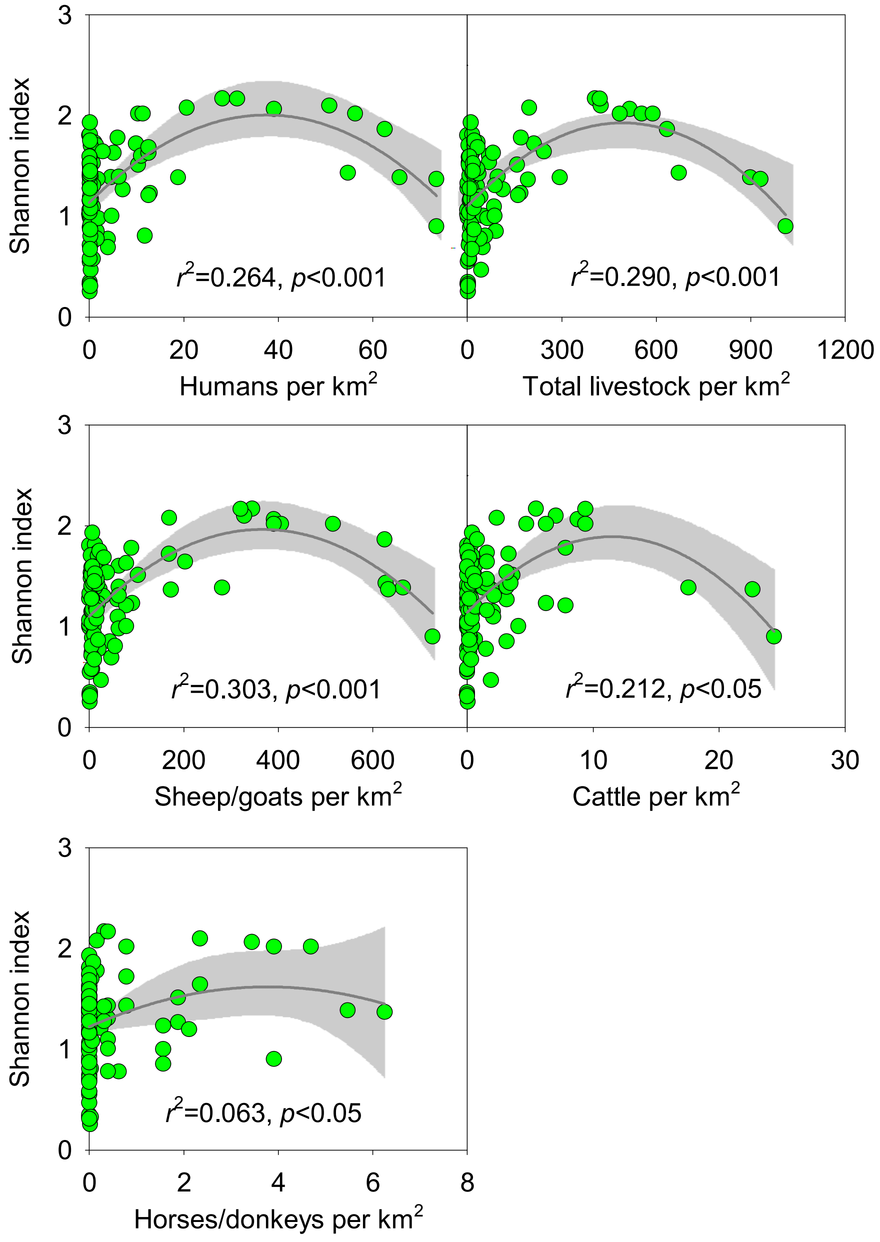

Figure S2

Relationships between Shannon-Wiener index and disturbance. The relationships are best described by non-linear regression (dark grey lines). Nonlinear patterns are derived by Loess smoothing (grey shade) with 95% confidence intervals.

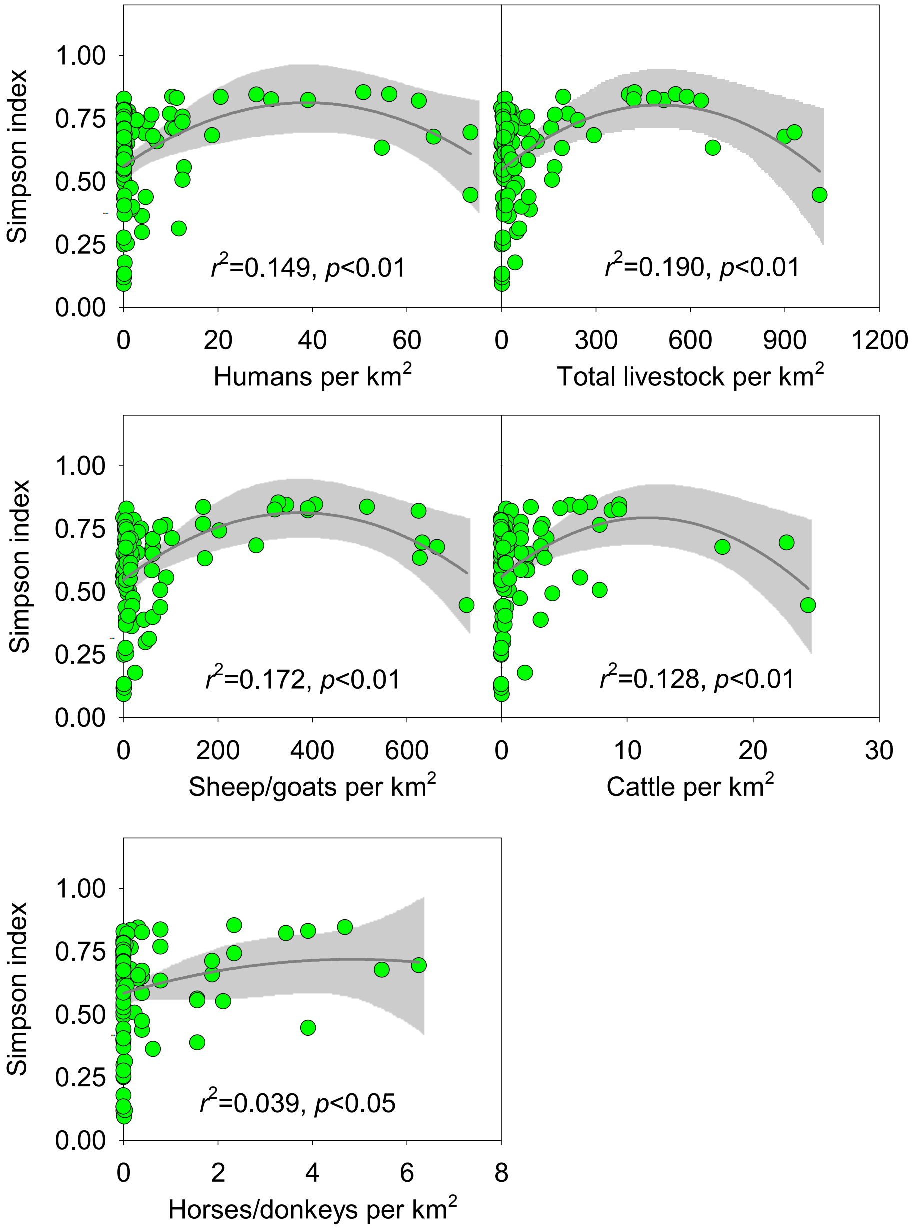

Figure S3

Relationships between annual plant biomass and disturbance. The relationships are best described by non-linear regression (dark grey lines). Nonlinear patterns are derived by Loess smoothing (grey shade) with 95% confidence intervals.

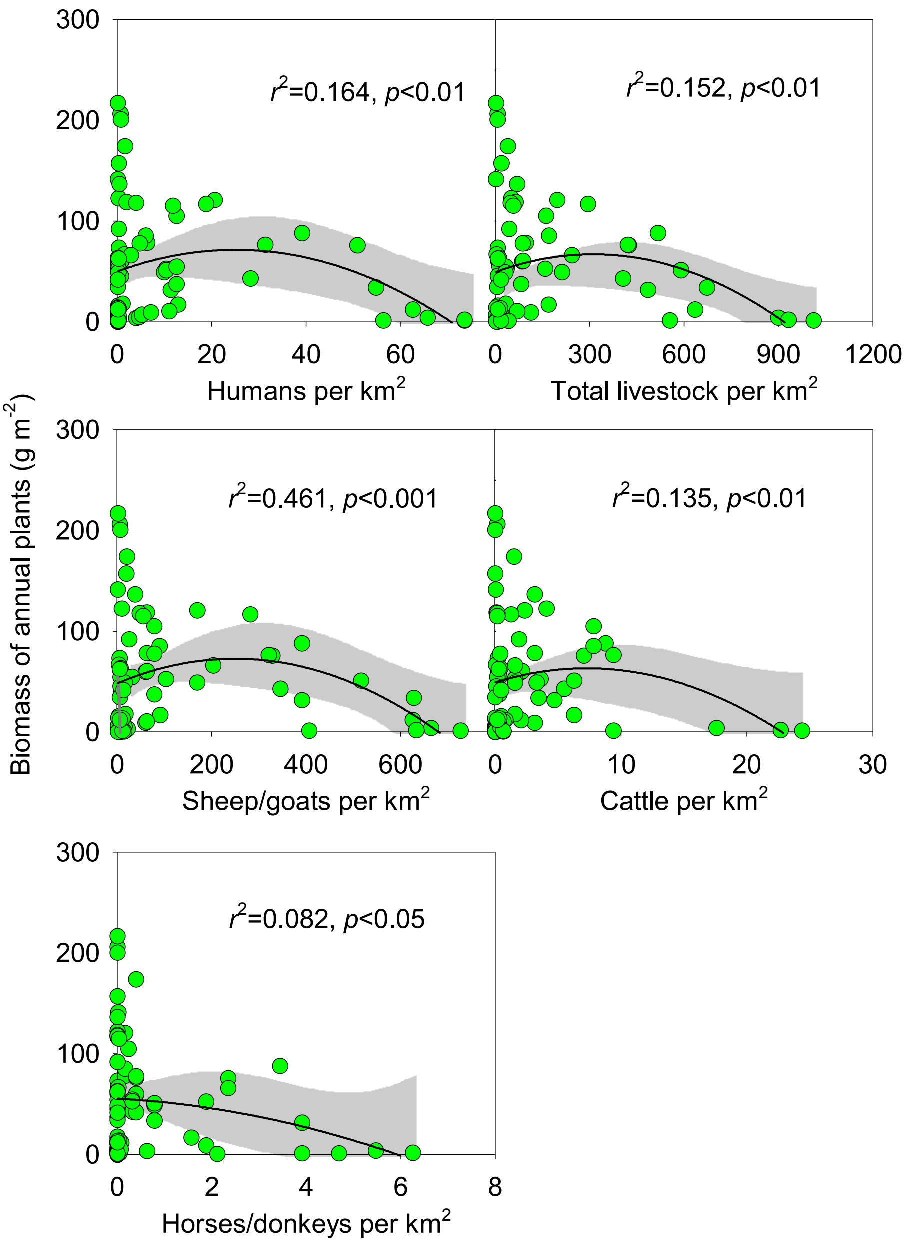

Figure S4

Relationships between unpalatable plant biomass and disturbance. The relationships are best described by non-linear regression (dark grey lines). Nonlinear patterns are derived by Loess smoothing (grey shade) with 95% confidence intervals.

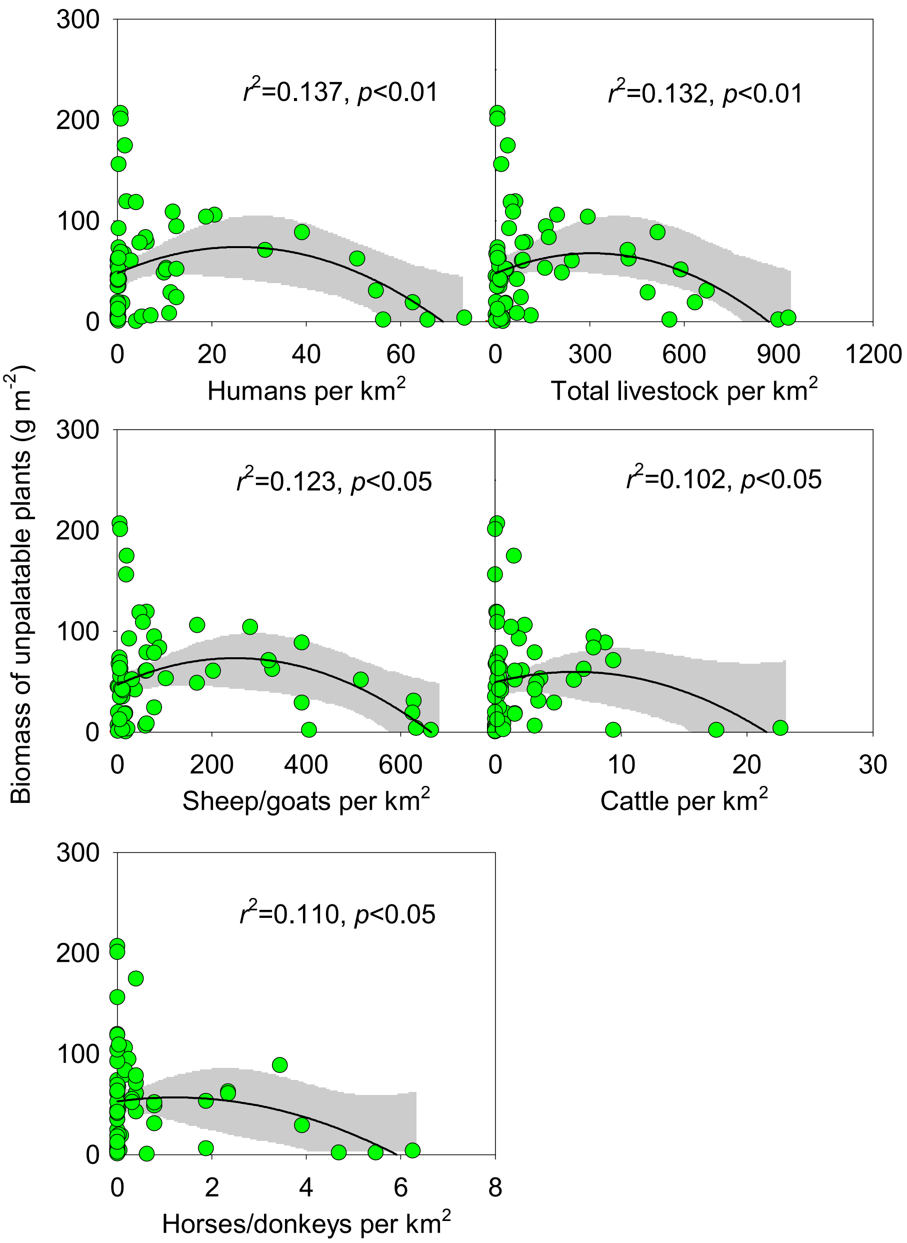

Supplement: Supplementary Information [file srep22132-s1.pdf]
